# The state of harm reduction in prisons in 30 European countries with a focus on people who inject drugs and infectious diseases

**DOI:** 10.1186/s12954-021-00506-3

**Published:** 2021-06-29

**Authors:** Heino Stöver, Anna Tarján, Gergely Horváth, Linda Montanari

**Affiliations:** 1 Institute for Addiction Research, Frankfurt University of Applied Sciences, Nibelungenplatz 1, 60318 Frankfurt am Main, Germany; 2 Hungarian Reitox National Focal Point, Széchenyi István tér 7-8, Budapest, 1051 Hungary; 3 European Monitoring Centre for Drugs and Drug Addiction, Praça Europa, 1, 1249-289 Lisbon, Portugal

**Keywords:** Prison, People who inject drugs, HIV, Hepatitis C, Hepatitis B, TB, Harm reduction

## Abstract

**Background:**

People who inject drugs are often imprisoned, which is associated with increased levels of health risks including overdose and infectious diseases transmission, affecting not only people in prison but also the communities to which they return. This paper aims to give an up-to-date overview on availability, coverage and policy framework of prison-based harm reduction interventions in Europe.

**Methods:**

Available data on selected harm reduction responses in prisons were compiled from international standardised data sources and combined with a questionnaire survey among 30 National Focal Points of the European Monitoring Centre for Drugs and Drug Addiction to determine the level of availability, estimated coverage and policy framework of the interventions.

**Results:**

Information about responses to health harms in prisons is limited and heterogeneous. Cross-country comparability is hampered by diverging national data collection methods. Opioid substitution treatment (OST) is available in 29 countries, but coverage remains low (below 30% of people in need) in half of the responding countries. Needle and syringe programmes, lubricant distribution, counselling on safer injecting and tattooing/piercing are scarcely available. Testing for infectious diseases is offered but mostly upon prison entry, and uptake remains low in about half of the countries. While treatment of infections is mostly available and coverage is high for human immunodeficiency virus (HIV) and tuberculosis, hepatitis B and C treatment are less often provided. Health education as well as condom distribution is usually available, but provision remains low in nearly half of the countries. Post-release linkage to addiction care as well as to treatment of infections is available in a majority of countries, but implementation is often partial. Interventions recommended to be provided upon release, such as OST initiation, take-home naloxone and testing of infections, are rarely provided. While 21 countries address harm reduction in prison in national strategic documents, upon-release interventions appear only in 12.

**Conclusions:**

Availability and coverage of harm reduction interventions in European prisons are limited, compared to the community. There is a gap between international recommendations and ‘on-paper’ availability of interventions and their actual implementation. Scaling up harm reduction in prison and throughcare can achieve important individual and public-health benefits.

## Background

In 2016, there were nearly 800,000 people in prison, including pre-trial detainees, in the 27 EU countries, Norway, Turkey and the UK (from now on EU-30) with national prison population rates varying from 51.4 people in prison per 100,000 inhabitants in the Netherlands to 244.6 in Turkey [1]. Criminal behaviour, drug use, lower socio-economic status and mental health conditions are interlinked factors that contribute to a higher risk of incarceration [2–4]. The prison population is seriously affected by drug problems: in 2016, one-sixth of people living in prison in Europe were incarcerated for drug offences [1]. Beside for offences against drug laws, people with drug problems are imprisoned for committing other types of crimes, in particular acquisitive crime to support their drug use [5]. Compared to the general population, people in prison are more likely to have ever used drugs in their lives or have experienced more severe drug-related problems [6, 7].

Among people who use drugs a high proportion of people who inject drugs (PWID) are imprisoned [8–10]. PWID carry a high burden of drug-related health consequences and risks [11–16] already before being imprisoned. Their incarceration further increases the risk of developing drug-related problems [17] including acquisition and transmission of human immunodeficiency virus (HIV), hepatitis B and C virus (HBV; HCV) [10, 18–24], which may impact on other risk groups they have contact with during imprisonment. Those who inject opioids are more prone to die from a fatal overdose inside prison and especially upon release [25–33]. Research underlines that after release injecting-related risk behaviour increases, which in the meantime also elevates transmission levels of infectious diseases in the community where they return to [21, 34, 35].

Prison settings are high-risk environments for virus transmission because of frequent risky behaviours, such as unsafe drug injecting, risky tattooing and unprotected sexual contacts; overcrowding; and limited or no access to appropriate diagnosis, care and treatment [36]. However, prisons could be a core setting to address the needs of hard-to-reach populations, such as PWID, with the provision of harm reduction, counselling, testing and treatment before they return to the community where many of them are yet again hard to reach and to enrol into treatment [34, 37].

Harm reduction interventions addressing drug-related infectious diseases and overdose deaths in the community, including opioid substitution treatment (OST), needle and syringe programmes (NSP), take-home naloxone (THN), and the testing and treatment of infectious diseases are supported by a large body of scientific evidence [38–43]. Although less studies on the effectiveness of these measures have been conducted inside prison than in the community, results are transferrable and European recommendations exist [10, 44–51]. Furthermore, equivalence and continuity of care are key principles guiding the implementation of health and social interventions in prisons [45, 49, 52, 53] and providing relevant services to people in prison benefits public health in general [45, 54, 55]. Despite this, harm reduction interventions have been implemented in prisons with a significant delay compared to community or have in some countries not been introduced at all [14, 56–59].

Information, education and counselling are the most widely implemented preventive and harm reduction measures in prisons, although they have been found insufficient to control and prevent specific drug-related harms, such as infectious diseases unless combined with other interventions [14, 45, 60]. Those comprise addiction treatment, including OST, distribution of sterile injecting equipment, the distribution of naloxone in prison and upon release; condom distribution, testing and linkage to infectious diseases care [14, 43, 45, 60–65]. The period of incarceration, especially for longer sentences, allows to provide infectious diseases treatment, including anti(retro)viral treatment of HIV and hepatitis infections, which are both effectively manageable in this setting [53, 60]. Testing and vaccination of people in prison upon entry and then at regular intervals—especially in case of those belonging to further risk groups such as men who have sex with men and PWID—are recommended in national and international guidance [45, 66–69]. An annual offer of infectious disease testing to PWID is recommended in community and prison settings [43, 70]. Overdose risk awareness and intervention training including naloxone distribution programmes have been evaluated as effective [71, 72]. NSPs have shown to be effective in preventing injecting-related harms; however, security measures in prisons can have serious hindering effects on their proper implementation [42, 45, 46]. There is limited evidence regarding the effectiveness of rinsing syringes with bleach in order to reduce their infectivity [45, 73]. Nevertheless, in a few prison systems bleach and disinfecting tablets are distributed to make up for the lack of availability of NSPs.

Data on the prevalence of drug use and infectious diseases in European prisons and information about interventions to address them is scarce. Previous studies on harm reduction interventions in European prisons have focused on more specific topics, such as prevalence, prevention or treatment of drug-related infectious diseases, more recently mostly on HCV [57, 74, 75] or were conducted a longer time ago, and their results may have become outdated [76]. Others have assessed the coverage of harm reduction measures but only in a limited number of countries [57, 77] or examined their compliance with international guidance [56, 78]. Several studies have acknowledged the gap between real-life implementation of harm reduction measures and their availability declared ‘on paper’, e.g. in policy documents [16, 79, 80], or found that even if harm reduction measures are available in prisons, their level of coverage and the quality of implementation may differ making European-level comparisons challenging [81].

The current paper aims to provide a fresh European overview on availability, coverage and policy framework of harm reduction interventions in prisons. The analysis is based on national, consolidated data collected via international agencies’ data sources covering 30 European countries and then validated and completed by the European Monitoring Centre for Drugs and Drug Addiction (EMCDDA) REITOX^1^ National Focal Points through a questionnaire survey [82]. The paper primarily focuses on prison-based interventions targeting injecting drug use-related health consequences and furthermore also includes interventions that are not directly addressing drug-related problems but are part of a package that can be provided to people in prison to prevent and control infectious diseases. Efforts have been made to go beyond official availability of several interventions and to assess their level of actual implementation and national coverage as well as to describe their place and framework in national policies.

## Methods

In the framework of the HA REACT^2^, a mapping survey was conducted regarding the prevalence of drug use, its health consequences, harm reduction interventions and its policy framework in prisons focusing on PWID and related harms such as infectious diseases and overdose. We covered all 30 countries which were members of the EMCDDA at the time of writing^3^: the 27 Member States of the European Union, Norway, Turkey and the UK (EU-30). They form together the EMCDDA’s Reitox network of national focal points and use a standardised reporting system developed by the EMCDDA which was the main data source for this survey. For this paper, a secondary analysis was conducted regarding data gathered via the mapping exercise focusing on interventions to provide a descriptive analysis and overview on the availability, coverage and policy framework of harm reduction interventions in prisons in the EU-30.

The method of mapping was based on a review of international agencies’ data sources and data collection from selected sources (Table 1) in the framework of a desk research followed up by a questionnaire survey among all 30 EMCDDA Reitox National Focal Points.

**Table 1:** Data sources* used during the desk research to compile the 30 National Profiles

Source	Availability
1. EMCDDA Prison Workbooks** 2017 (2016 data)	Restricted
2. EMCDDA Special Issues on Prison in 2011	Restricted
3. EMCDDA Statistical Bulletin 2017 (2016 data)	Public
4. EMCDDA Standard tables and questionnaires** ST10; ST24; SQ27 P1 (data on 2016 or before retrieved in 2017/2018)	Restricted
5. ECDC Dublin Declaration Questionnaire 2018 (2017 data) (data of preselected variables were provided by the ECDC)	Restricted
6. Council of Europe Space Project 2018 (2016 data)	Public

^*^Only harm reduction intervention-related sources are listed as drug use and HIV/HCV prevalence is not covered in this paper

^**^EMCDDA drug-related thematic Workbooks and standard tables/questionnaires are annual, standardised reporting tools based on common European methodological guidelines, reporting framework and definitions used in the EU-30 to ensure data harmonisation, aggregation and comparability at EU level. Reporting quality of each country is annually evaluated by the EMCDDA. In the Prison Workbook qualitative information is provided on the prison and drugs situation at national level and quantitative data on prevalence of drug use among people in prison and selected drug-related interventions inside prisons

The data collection and analytical process had 4 phases:

Phase 1: Desk research (2017 December–2018 May)

A review was conducted to collect and assess all available information and data sources about drug use, HIV/HCV prevalence and harm reduction interventions in prisons in the countries covered. After that—according to data access, the level of data availability and detailedness of information certain sources were selected for the mapping survey that are listed in Table 1.

The primary sources of information (Table 1) were the Prison Workbooks and standard tables provided by the Reitox National Focal Points of the EMCDDA. The National Focal Points’ reporting tools are considered the best available data on this topic that are collected according to a unified methodology and case definitions of the EMCDDA, reported in the same structure and delivered to the EU’s drug agency in English language. Although the EMCDDA publishes the main findings based on the countries’ reporting tools, those detailed sources are otherwise restricted. All Reitox National Focal Points were contacted, and they gave consent to have their data utilised. As a result of the Reitox reporting mechanism, all data sent to the EMCDDA are scrutinised, consolidated and gone through a national administrative approval process, and qualitative information originating from this source is triangulated among different national-level sources (e.g. ministries and related institutions, non-governmental organisations (NGOs), independent researchers). However, in the same time, limitation of this data source is that although at European level standardised information is collected, at national level it depends on the National Focal Points and the available data sources how and from which sources they compile the requested information that can include expert estimates, national prison registries, grey data of external prison service providers, independent research projects as well as well-designed studies.

Phase 2: Building the national profiles (2018 May–2018 Aug)

A set of common and feasible (based on data availability) core variables for analysis were identified on the basis of data sources. Six research domains were set up into which variables were linked: 1. general prison data; 2. Drug use/Injecting drug use among people in prison 3. Infectious diseases among people in prison 4. Harm reduction responses in prison 5. Testing, Vaccination and Treatment of infectious diseases in prison 6. Framework and Strategies for harm reduction in prison. We built 30 ‘national profiles’ under the six domains to which 103 variables were linked (all variables are enlisted in the comprehensive European Mapping Report of Harm Reduction Interventions in Prisons (EMR), hyperlink to the report is placed under section ‘Availability of data and materials’).

Phase 3: Questionnaire survey among national prison experts (2018 Aug–November)

National Profiles were formatted as prefilled questionnaires including the information extracted during the desk research. Afterwards the Heads of the Reitox National Focal Points of the EMCDDA in all countries were contacted and sent the respective ‘national profiles’. Heads of Focal Points could involve prison experts working with the Focal Points to complete the questionnaire. We asked them to confirm or update the prefilled data or add data if information was not available at sources. Regarding questions on coverage—we asked them to assess them if data could not be retrieved or calculated from sources. That data consolidation process was complemented by bilateral, oral consultations when needed. After the questionnaire survey and bilateral consultations with the 30 countries, we compiled the European mapping report and database (See: EMR). It was indicated when information was not available neither by the desk research nor by the national expert consultation process for analytical purposes.

Phase 4: Secondary analysis of data domains related to harm reduction interventions and policy framework

For this paper, we only analysed variables (*n* = 67) linked to the last 3 domains that refer to interventions and responses in the prison setting that are listed in Table 2.

**Table 2:** List of analysed variables on drug-related harm reduction interventions and its policy framework

Harm reduction responses in prison	Testing, Vaccination, Treatment of infectious diseases in prison	Framework and Strategies for harm reduction in prison
Screening of people in prison for drug-related problems upon entry	HIV testing available	Responsible institution for prison health/prison structure
OST available	HIV testing rate (%) among people in prison last year	external agencies (incl. NGOs) included in harm reduction service provision
OST coverage 1.—% of prisons where available	HIV testing coverage (last year) estimated if rate cannot be calculated	Strategy document for drug-related responses in prison available
OST coverage 2. % of people in prison in need receive OST	HCV testing available	Guidelines/strategy for drug-related responses in prison where
Number of inmates receiving OST	HCV testing rate (%) among people in prison last year	Guidelines/strategy for harm reduction in prison available
Dominant type of OST medication provided in prisons	HCV testing coverage (last year) estimated if rate cannot be calculated	Guidelines/strategy for harm reduction in prison where
OST Detoxification available	HBV testing available	Guidelines/strategy for testing/treatment of infectious diseases in prison available
OST continued for people in prison already in OST before entering prison available	HBV testing rate (%) among people in prison last year	Guidelines/strategy for testing/treatment of infectious diseases in prison where
OST initiated after entering prison available	HBV testing coverage (last year) estimated if rate cannot be calculated	Guidelines/strategy for harm reduction measures upon release available
OST initiated before release available	TB testing available	Guidelines/strategy for harm reduction measures upon release where
NSP available	TB testing estimated coverage last year	Equivalence of care
NSP coverage 1.—% of prisons where available	Vaccination for HBV available	Continuity of care
NSP coverage 2.—% of people in prison in need receive NSP	HIV post-exposure prophylaxis available	
Distribution of bleach available	Antiretroviral therapy for HIV available	
Estimated coverage of bleach distribution: % of prisons where available	Antiretroviral therapy for HIV estimated coverage	
Condom provision available	Antiviral therapy for HCV available	
Estimated coverage of condom promotion and distribution programmes in prisons, % of prisons where it is provided	Antiviral therapy for HCV estimated coverage	
Lubricants provision available	Antiviral therapy for HBV available	
Information and education on drug-related health risks (in general) available	Antiviral therapy for HBV estimated coverage	
Health education to prevent overdoses during imprisonment available	TB treatment available	
Health education (as prevention) on drug-related infectious diseases available	TB treatment coverage	
Health education on drug-related infectious diseases coverage: % of people in prison receive it	Linkage to HIV care upon release	
Health education (as prevention) on sexually transmitted diseases available	Linkage to HCV care upon release	
HIV-related health promotion or behaviour change programmes in prisons coverage		
Information and education on risks of tattooing and piercing available		
Training on safer injecting available		
Harm reduction/addiction service provided to people in prison with drug problem upon release available		
Health education to prevent overdoses upon release available		
Distribution of naloxone upon release available		

Time frame: Data collected through the desk research phase referred to 2016 or latest available data (except for a few variables retrieved from ECDC where data referred to 2017 or latest available before 2017.) During the questionnaire survey, we asked the countries to check the provided numeric data and/or to add 2016 data or latest available. 2017 data (or latest available) were asked concerning availability and coverage of services. Thus, the analysis presented in the paper reflects the situation in 2016/2017. As an exception 2018 update on OST availability was added later although this is out of the scope of the monitored period.

Data level per country: The collected data refer to the national level, which was preferred in the request for data collection over regional data even if the latter were newer. Only for the UK data were collected in 4 units for administrative reasons, however, they were merged for the present analysis (as explained in footnotes). In the paper we aim to give a European overview and almost no data by country are presented. Country-level data can be consulted through the mapping report (see: EMR).

Three stages of imprisonment: We identified 3 stages of imprisonment: upon entry, during imprisonment and upon release that are relevant and distinctive in case of provision of harm reduction interventions and are considered as important stages in the continuity of care. In the results section interventions related to the first two stages are described together, while interventions upon release are presented in a different section.

Data on coverage range: Coverage range refers to the number of prisons covered with an intervention, or the number of people in prison covered with an intervention or the number of people in prison in need of an intervention. This is specified in the results section for each intervention. In all but one variable, the following thresholds were set up for coverage ranges: no coverage; low coverage: below 30%; medium coverage: 30–60%; high coverage 61–95%; full coverage: above 95%. In one case—OST coverage in prisons—due to the original data source the following categories were applied: full: above 75%; high: 50–75%; medium: 25–50%; low: below: 25%; no coverage.

The estimate of coverage range for testing uptake was calculated on the basis of testing rate; if not available, the coverage range was given by expert estimate.

Valid data for analysis: In the analysis, we considered valid those answers where information was provided. Countries providing no information or ‘do not know’ for a given variable were excluded from the analysis; the number of countries with no information is indicated.

## Results

### Availability and coverage of harm reduction interventions upon entry and during imprisonment

#### Assessment of drug-related problems upon entry

**Fig. 1:**
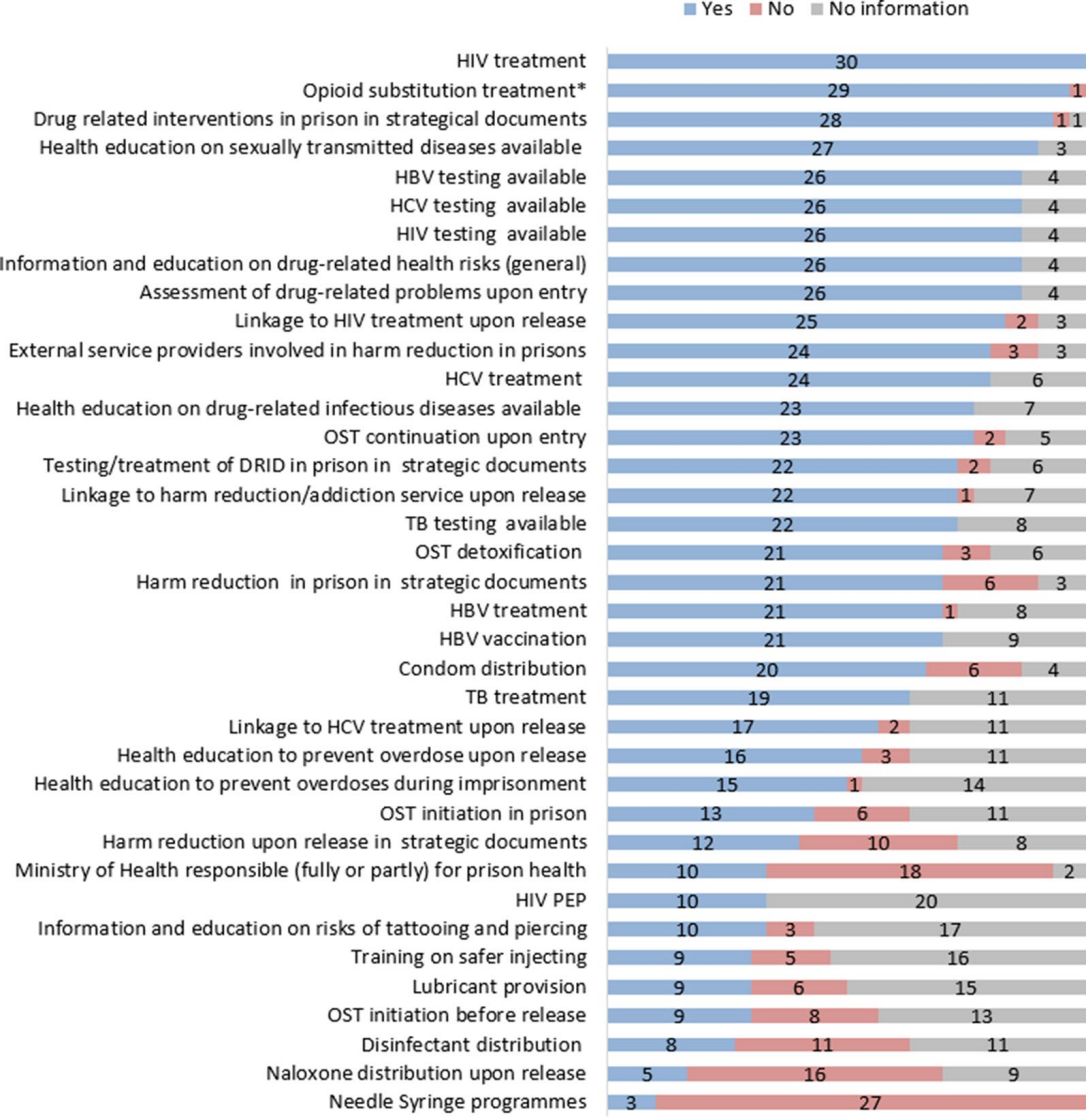
Policy framework for and availability of harm reduction interventions in prisons in the EU-30 in 2016/2017. *OST availability refers to 2018, when Lithuania also introduced this intervention

In the 26 reporting countries with a valid answer (4 with no information), people in prison are screened for drug-related problems upon entry; however, it is usually part of a general health/mental health assessment. Spain specified that the evaluation for injecting related risk behaviours is also part of the upon entry assessment process (Fig. 1).

#### Interventions targeting prevention of overdose and infectious diseases

**Fig. 2:**
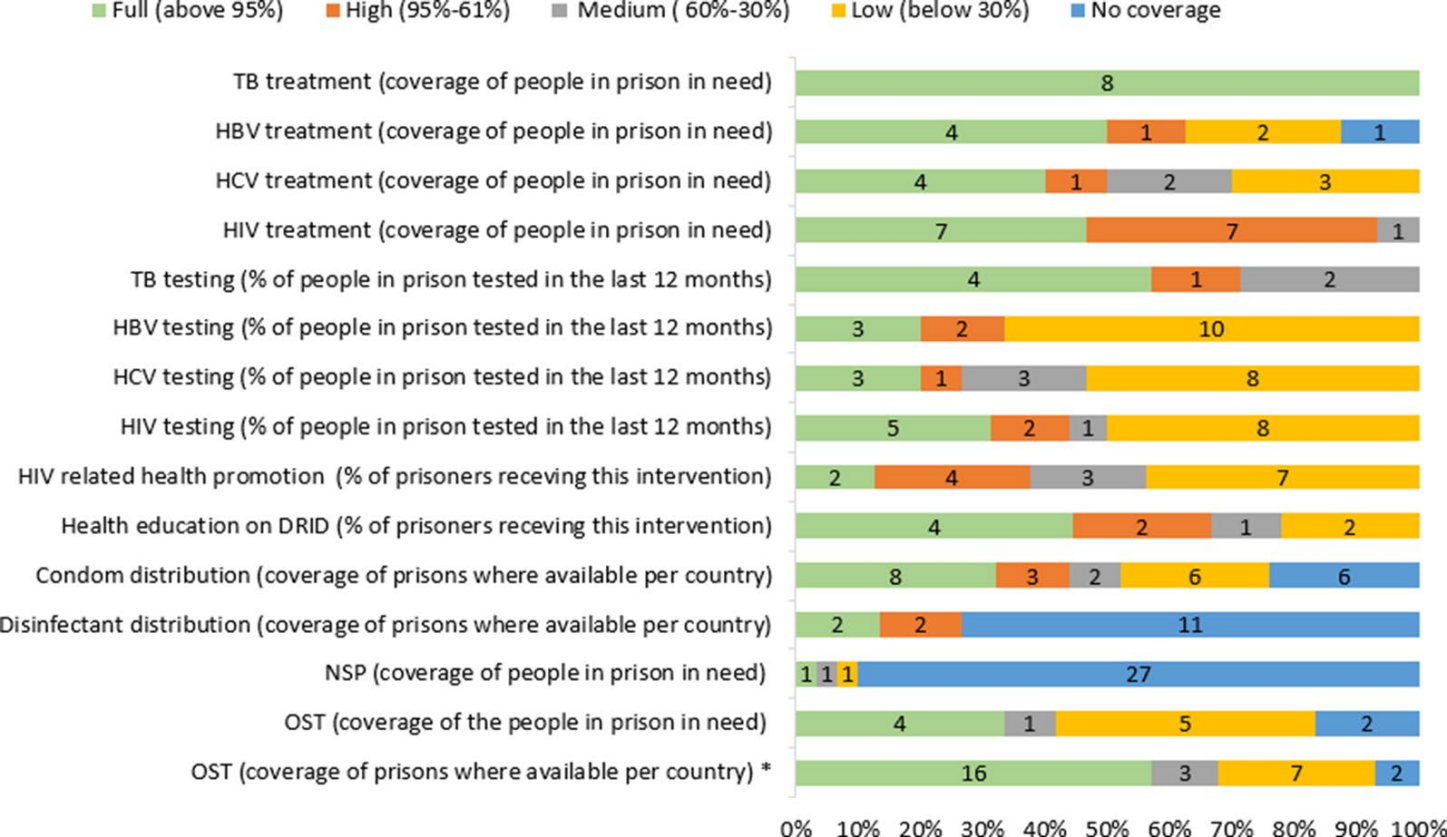
Coverage of selected harm reduction interventions in prisons in the EU-30 in 2016/2017 (no. of countries) (countries with no information on availability and/or coverage are not presented in this figure) *OST—coverage of prisons where available per country had different thresholds for the categories: full: above 75%; high: 50–75%; medium: 25–50%; low: below: 25%

OST is available in all but 1 country (Slovakia) in prisons among the monitored countries (Fig. 1). The latest country introducing it was Lithuania in 2018.^4^ Coverage of OST (based on the number of prisons where OST was available) varied greatly in 2016/2017 among the 28 reporting countries: The proportion of prisons providing OST was over 75% in 16 countries, 25–50% in 3 countries, while less than 25% in 7 countries. In Lithuania in 2017 it was not yet provided despite perceived need, while in Slovakia it was not provided and reportedly there was no perceived need (Fig. 2).

**Fig. 3:**
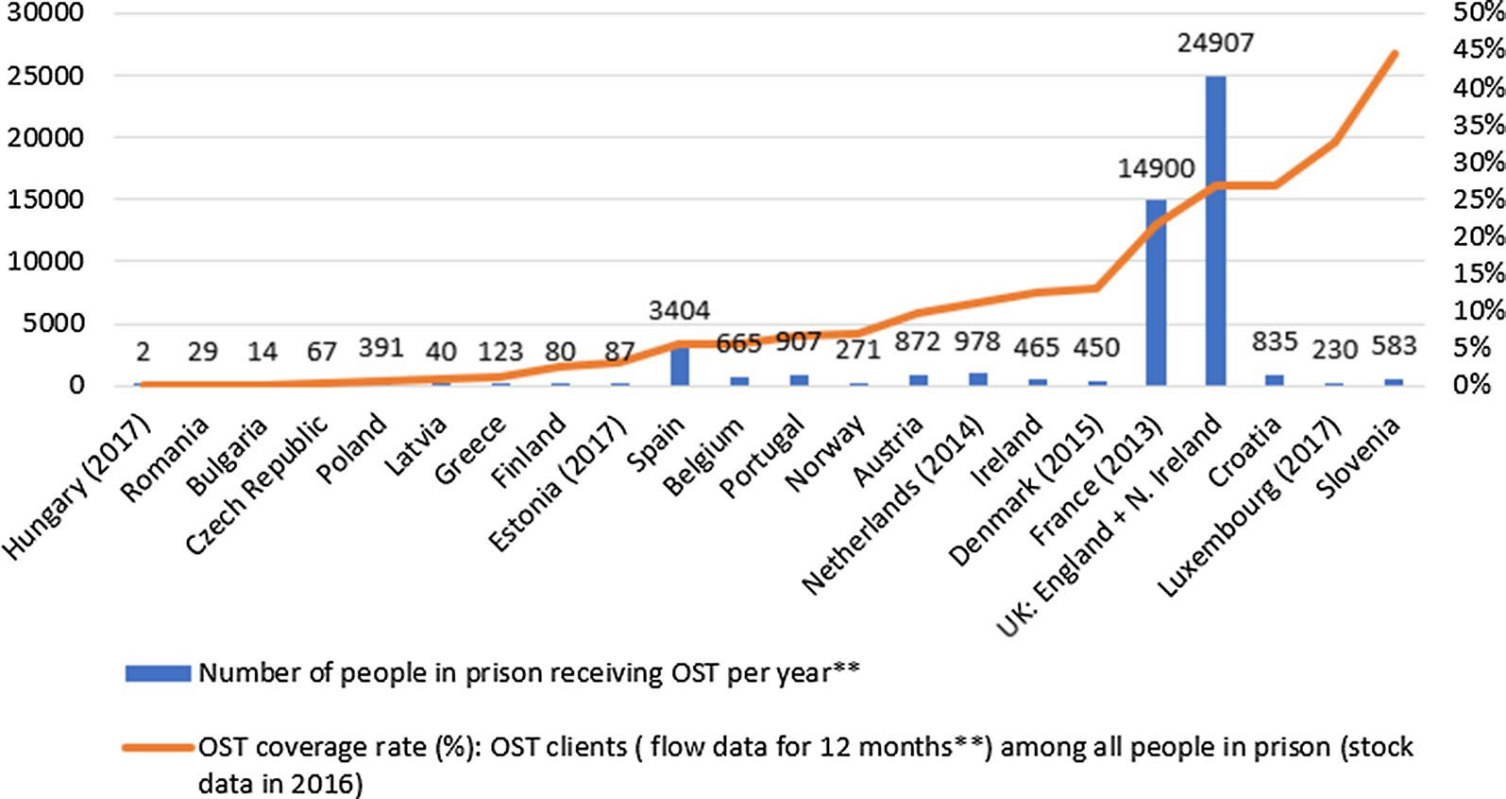
Number of people in prison receiving OST in 2016 and proxy* coverage of OST among total prison population in 22 European countries. If year of data is different for N of OST clients it is indicated in the figure. **OST client data was not flow but stock data in case of Portugal, Norway, Belgium, Ireland. Proxy coverage was calculated on the basis of number of OST clients and total prison population data available at SPACE statistics for 2016 (stock data). *It is proxy as the denominator is all people in prison during the reporting year instead of people in need in prisons during the reporting year, as the latter is not available thus it is not sensitive data in terms of the level of demand

In the 22 countries with data on the national annual number of OST clients in prison, 448 891 people were incarcerated in 2016 (stock data), while the aggregated number of people in OST in the respective countries (flow data for one year in 18 countries and stock data for a given a year in 4 countries) was 50,300 in 2016. The number of OST clients in prison ranged from 2 persons in Hungary to 24,907 in the UK^5^ (Fig. 3). Proxy^6^ coverage rate of people in OST among all people in prison ranged from 0,01% in Hungary to 44,6% in Slovenia (Fig. 3). In 5 countries, this rate remained under 1%, in 9 countries between 1 and 10%, in 6 countries between 10 and 30%, while in 2 countries between 30 and 45% (Fig. 3).

Ten countries assessed coverage of OST in terms of the number of people imprisoned in need: 4 countries assessed it full (95–100% of people in prison in need), 1 country medium (30–60%), while 5 countries low (less than 30%) (Fig. 2).

Out of the 28 countries where OST was available in prisons in 2017, 23 countries reported on the type of OST medication utilised in most cases. Methadone is the predominant medication used in 17 countries, while buprenorphine [2] or the buprenorphine–naloxone combination [4] is used primarily in six countries.

Twenty-one countries reported that OST detoxification is available in prisons, while 3 countries reported no access to such service (6 countries no information). Twenty-three countries confirmed that OST can be continued for people in prison already in OST upon entry; it is not possible in 2 countries where OST is not provided (5 countries: no information). OST can be initiated after entering prison in 13 countries, while 6 countries reported that it was not possible, while no information was available in the case of 11 countries (Fig. 1).

Prison-based needle and syringe programmes (PNSPs) are only available for people in prison in Spain, Germany and Luxembourg (Fig. 1); in Spain and Luxembourg other sterile drug injection equipments are also provided. In Romania, PNSPs operated in several prisons for some time but have been discontinued after external funding stopped. France is planning to implement NSP in prisons, other sterile injecting paraphernalia is already distributed. In the Netherlands, PNSPs are not implemented as there is reportedly no indication of injecting drug use in their prisons. In Germany, a single programme exists in a women’s prison in Berlin out of 181 prisons in total; thus, coverage is assessed low in terms of the number of prisons where available and in terms of the number of people in need accessing that service. In Luxembourg and Spain, the intervention is available in nearly all prisons (full coverage: 95–100% of prisons). However, coverage in terms of reaching people in need was evaluated differently, as medium level by Spain and full by Luxembourg (Fig. 2). The most frequently reported reasons for not providing such services in the remaining countries are the prohibition on drugs in prison and the safety of the prison staff.

Distribution of disinfectants (mainly bleach) to clean drug-injecting equipment is available in eight countries.^7^ Eleven countries did not provide data and 10 countries reported that it is not available in their prisons, while the Netherlands reported no relevance due to no injection in prisons (Fig. 1). Coverage data (regarding the percentage of prisons where the service is available) was reported by 4 countries and was estimated to be full in 2 of them, while high in the other two (Fig. 2).

Condom distribution programmes for people in prison are available in 20 countries, in 6 countries it is not provided, while 4 countries did not provide information on this. Lubricants are provided in 9 countries, while at 6 there is no such intervention, in case of 15 countries information was not available (Fig. 1).

In terms of condom promotion and distribution programmes, information on coverage (percentage of prisons where the service is provided in a given country) was provided from 25 countries. Full coverage is available in 8 countries, high coverage in 3, medium coverage in 2, while 6 countries reported low coverage of such intervention. In 6 countries, these programmes do not exist; thus, there is no coverage of such intervention^8^ (Fig. 2).

#### Information, education, training on harms and safer behaviour

Health education on sexually transmitted diseases, health education on drug-related infectious diseases (DRID) and information and education on drug-related health risks in general were available in all reporting countries that had information on this, 27, 23 and 26, respectively. Training on safer injecting was reported to be available in 9 countries, 5 countries reported it was not available, while 16 respondents had no information on this. Information and education on risks of tattooing and piercing is provided in 10 countries, while it is not provided in 3, 17 countries did not have this information. Health education to prevent overdoses during imprisonment was provided in 15 countries, while it was not provided in 1 country, 14 respondents did not have information (Fig. 1).

Coverage of health education on DRID regarding the proportion of all people in prison receiving such intervention was estimated to be full in 4 countries, high in 2 countries, medium in one country while low in 2 further countries among the 9 reporting countries. Coverage of HIV-related health promotion or behaviour change programmes—regarding people in prison receiving the intervention—was reported by 16 countries: estimated to be full in 2 countries, high in 4 countries, medium in 3 countries while low in 7 further countries (Fig. 2).

#### Vaccination, testing and treatment of infectious diseases

**Fig. 4:**
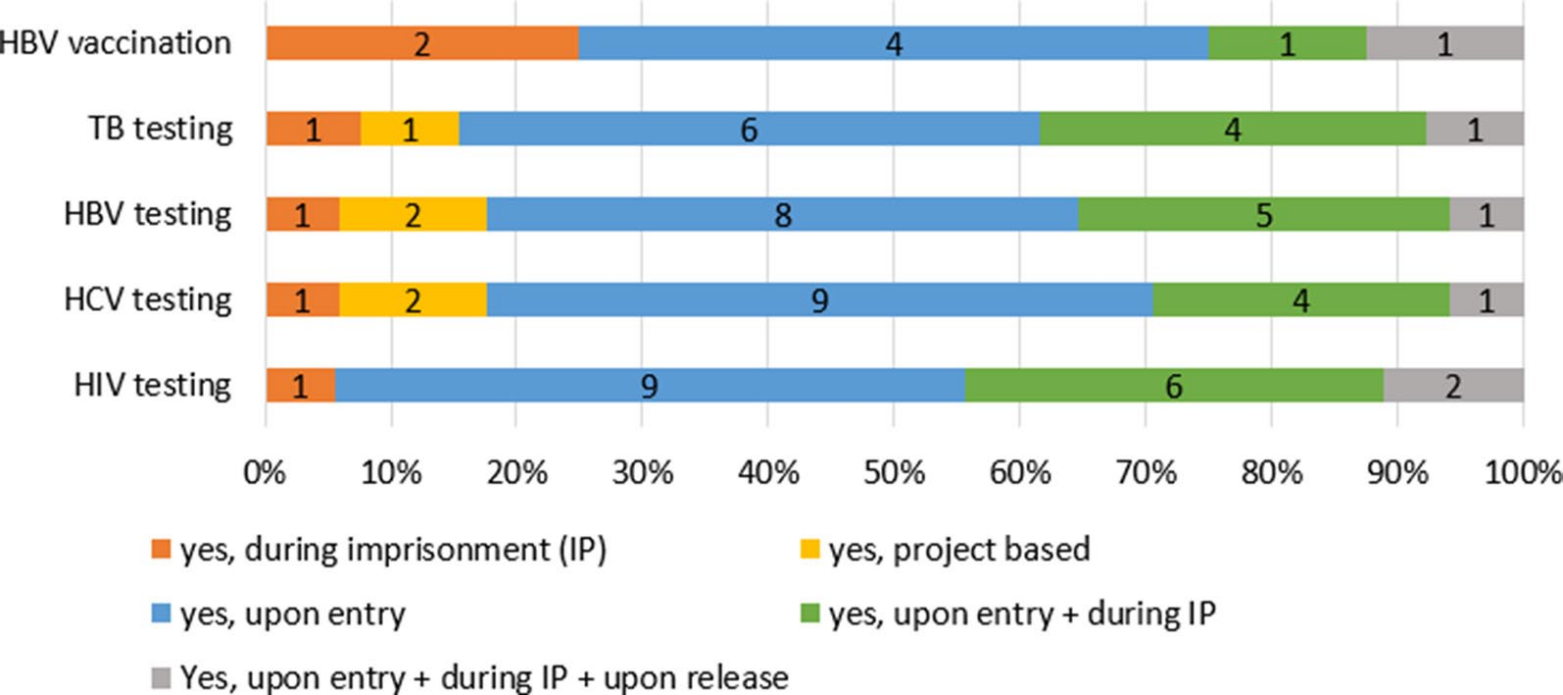
Testing and vaccination by stages of imprisonment when it is offered in prisons in the EU-30 in 2016/2017 (*n* = reporting countries). *Countries with no information on availability of testing and vaccination and/or detailed information on its implementation are not presented in this figure*

Vaccination against hepatitis B virus (HBV) is available in 21 countries, while one country reported that is not provided in their prisons (Fig. 1). Eight countries reported at which stage of imprisonment the vaccination is offered: in 4 it is offered upon entry, 2 countries provide it only during imprisonment, while 1 country offers it upon entry and during imprisonment and another covers all 3 stages of imprisonment (Fig. 4). All 10 countries with a valid answer report on the availability of HIV prophylaxis (Fig. 1).

All the 26 countries with a valid answer provide HIV, HCV and HBV testing for people in prison. Treatment for HIV, HCV is available in all countries with a valid response 30, 24, respectively, while HBV treatment is available in all but one among 22 reporting countries (Fig. 1).

For HIV 18 countries shared information in which phase the testing was offered. Half of the countries [9] only offer HIV testing upon entry. One country provides such service only during imprisonment for people in drug treatment. Six countries provide this intervention upon entry and also during imprisonment. HIV testing is offered upon entry during imprisonment and also upon release in 2 countries (Fig. 4).

**Fig. 5:**
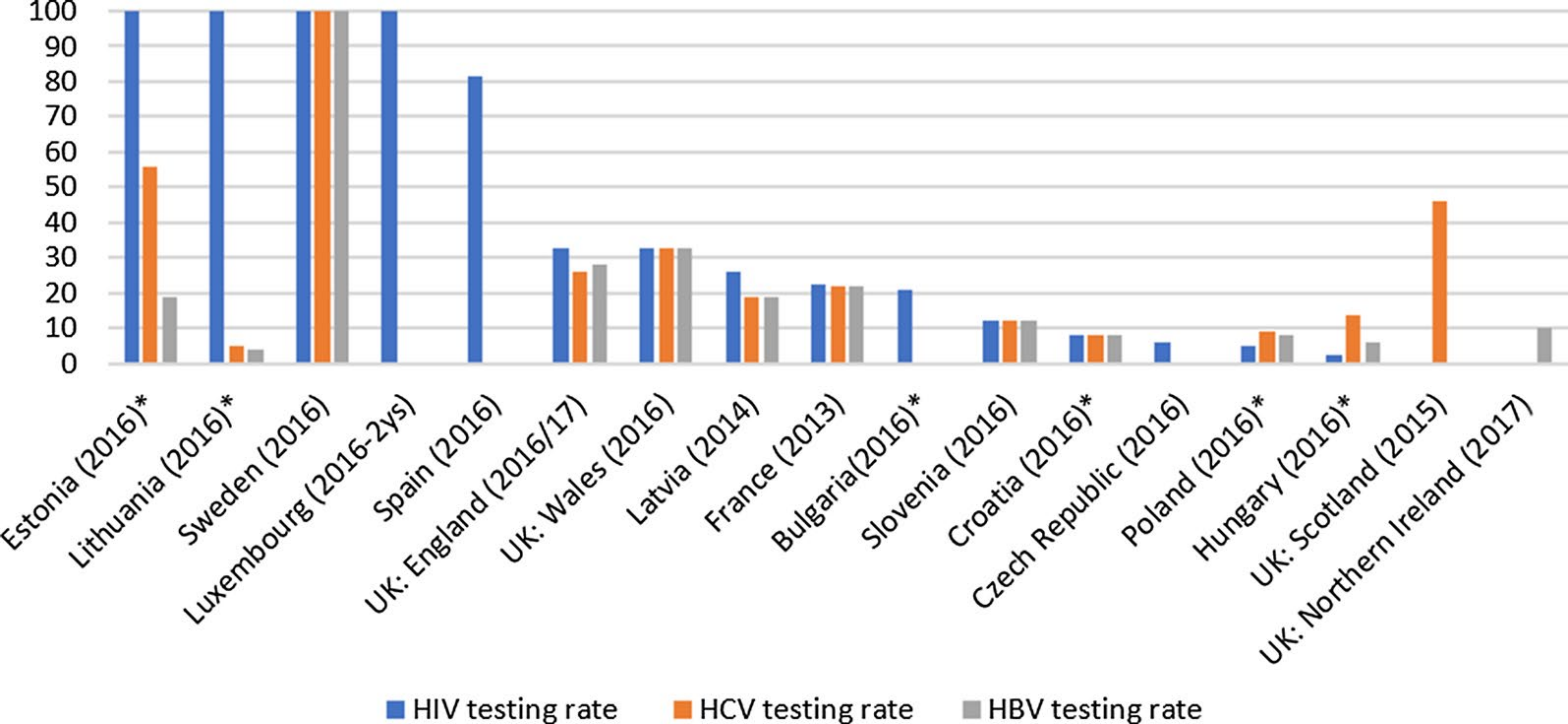
Proportion (%) of people in prison tested for infectious diseases in the last 12 months (2013–2017) in 14 European countries. *Data comparability across countries are limited: testing rate was calculated on the basis of number of tested people in the last 12 months (flow data) reported in EMCDDA Workbooks for 2016 and total prison population data available at SPACE statistics for 2016 (stock data) if testing rate per se (percentage, %) for the last 12 months was not found at sources during the desk research or not reported during the questionnaire survey; UK presented in 4 parts due to separate data reporting; Information per country is presented only for those viruses in case of which data were available

Data on HIV testing rates among people in prison in the last year were available in 14 countries, which ranged between 100% and 2.3%. Testing rates were above 80% in 5 countries, between 33 and 21% in 4, and between 12% and 2,3% in 5 countries (Fig. 5).

HIV testing coverage range estimation was available from 16 countries according to which 5 countries reported full coverage (> 95% of all people in prison tested last year), 2 high coverage (95–61%), 1 medium (60–30%), while 8 low coverage (< 30%) of HIV testing among people in prison in the last year^9^ (Fig. 2).

Coverage of HIV treatment was reported to be full (> 95% of people in prison in need are in treatment) in seven and high (95–60%) in another 7 countries and medium (60%—30%) in 1 country out of 15 where this information was available. (Fig. 2).

Regarding HCV, 17 countries shared information in which phase the testing was offered. Nine countries offer HCV testing upon entry, among them one country also offers Fibroscan test upon admission. One country provides such service only during imprisonment for people in drug treatment. Four countries provide this intervention upon entry and also during imprisonment, while in 2 countries provision of HCV testing is project-based. HCV testing is offered upon entry, during imprisonment and also upon release only in 1 country (Fig. 4).

Data on HCV testing rates among people in prison in the last year were available in 11 countries, which varied between 100 and 5% (Fig. 5). Testing rates were above 80% in 2 countries, between 56 and 22% in case of another 3 countries, while between 19 and 5% in 7 countries. As for coverage range among the 15 reporting countries, full coverage of HCV testing in the last year was estimated in 3 countries, high coverage in 1 country, medium coverage in 3^10^ countries, while coverage was low in 8 countries (Fig. 2). Of the 10 countries providing an estimation of coverage of those who need HCV treatment, 4 reported full, 1 high, 2 medium, while 3 countries rated coverage low (Fig. 2).

In terms of HBV, 17 countries shared information on which phase testing is being offered. Eight countries offer HBV testing upon entry. One country provides such service only during imprisonment for people in drug treatment. Five countries provide this intervention upon entry and also during imprisonment, while in 2 countries provision of HBV testing is project-based. HBV testing is offered upon entry, during imprisonment and also upon release only in 1 country (Fig. 4).

HBV testing rate among people in prison in the last year ranged between 4 and 100% (11 countries) (Fig. 5). Testing rates were above 80% in 2 countries, while between 33 and 22% in 2 countries, and between 19 and 4% in 7 countries.^11^ Estimates on coverage range of HBV testing in the last 12 months were available in 15 countries. Regarding coverage range: full coverage was reported in 3 countries, high coverage in 2 countries and low coverage in 10^12^ countries (Fig. 2).

Eight countries provided information on coverage: it was estimated to be full in 4, high in 1, low in 2 countries, while it is not provided thus there was no coverage in 1 country (Fig. 2).

As for tuberculosis (TB), testing is available in all 22 countries with a valid answer (Fig. 1). Thirteen countries specified its framework: in 6 it is available upon entry, in 4 countries it is available upon entry and then during imprisonment. One country only provides this for people in drug treatment, in one country it is available in the framework of projects, while one country provides it in all 3 stages of the prison stay (upon entry, during imprisonment and upon release) (Fig. 4).

Estimation on coverage range of TB testing was available only in 7 countries, 4 countries reported full coverage, one country reported high coverage, while 2 countries medium coverage (Fig. 2).

Treatment for tuberculosis is also available in prisons in all 19 reporting countries. In all the countries reporting TB data, treatment coverage of people in need is estimated to be full (8 countries) (Fig. 2).

### Interventions upon release, linkage to care in the community

#### Interventions to prevent overdose upon release and linkage to addiction care

Harm reduction or addiction services before release and linkages to community services are provided to people in prison with drug problems in 22 countries, it is not available in 1 country (however some information is provided), while no information could be gained for 7 countries. In terms of content, there is a great variation across countries: in France a designated person coordinates continuity of care in the case of OST. In Spain, OST or other types of addiction treatment are organised before the release of people with drug problems. In Germany, in some prisons a higher dose of opioid substitution medication is provided before release and counselling takes place on risks. Croatia provides this support for people in prison in collaboration with external public health agencies and NGOs. In 9 countries, OST can be initiated before release, in 8 countries it is not possible (no information: 13) (Fig. 1).

Naloxone distribution is available in 6 countries, 15 countries reported that it is not available, while information could not be retrieved in case of 9 countries (Fig. 1).

Naloxone distribution upon release in England, Germany and Norway has been available in the framework of projects, while it is routinely available in all the other parts of the UK (Wales, Scotland, Northern Ireland), Estonia and France. In the Netherlands, naloxone is available in prisons in general (not explicitly upon release) in case of emergency; however, there are no more specific data on the use of this intervention.

Health education to prevent overdoses upon release is available in 16 countries, not available in 3 countries, while no information was available on this topic in 11 countries (Fig. 1).

#### Testing upon release and linkage to infectious disease care

Testing upon release is only available sporadically, 2 countries reported to provide testing upon release for HIV, only 1 country provides testing for HBV, HCV and TB (Fig. 4).

The majority [25] of the countries stated that linkage to HIV care upon release was available: This service was partially available in 15 countries and fully available in 10 countries. Only two countries stated that a referral system was not in place, 3 countries did not provide information about the opportunity. Linkage to HCV treatment is fully available in 9 countries^13^ and partially available in 8 countries, whilst in 2 countries it is reportedly not available, 11 countries did not provide this information (Fig. 1).

#### Institutional and policy framework for prison health and harm reduction interventions

The overall public authority that is responsible for the implementation of health-related responses in prisons is the Ministry of Justice in 16 countries and the Ministry of Interior in 2 countries. Ministry of Health is responsible alone in 6 countries, while in collaboration with the Ministry of Justice in 4 countries (Fig. 1).

External service providers including NGOs are involved in providing harm reduction interventions in prison to a large extent in 2 countries, to some extent in 22 countries while they are not involved in 3 countries among the 27 countries providing valid answer to this question (Fig. 1).

Among countries with a valid answer, strategic documents for drug-related responses in prisons in general were available in 28 countries and were not available in 1 country (Fig. 1). Prison-based harm reduction interventions, testing and treatment for infectious diseases, and harm reduction upon release were included in 21, 22 and 12 countries’ strategic documents or guidelines, while it was not included in 6, 2, and 10 countries’ documents, respectively.

Drug-related interventions in the prison setting in general were mentioned both in drug and prison strategic national documents in 6 countries; both in drug and health strategy documents in 5 countries; while both in prison and health documents in 3 countries. In 5 countries, this topic was covered by all three related domains: drug, prison and health strategic documents. In 7 countries, it was only covered by 1 type of strategic document, which was in 6 cases a drug-related strategic document, and in 1–1 case prison or health-related document. Drug-related interventions in prisons are not covered in any strategic documents in one country (No information: 1 country).

Harm reduction interventions specifically in the prison setting were mentioned both in health and prison strategic documents in 1 country; both in drug and health strategy documents in 3 countries. In 3 countries, this topic was covered by all three related domains: drug, prison and health strategic documents. In 14 countries, it was only covered by 1 type of strategic document, which was in 5 cases a drug-related strategic document, in 5 cases health-related document, while in 4 cases a prison-related document. Explicitly harm reduction interventions in prisons are not covered in strategic documents in 6 countries (No information: 3 countries).

Nineteen countries indicated that the principle of continuity of care is stated in their written strategic documents and guidelines referring to the prison setting and that it mostly implemented in practice; 2 countries stated that it is set in documents, however not really implemented; in 3 countries it is not stated, but partly implemented (no information: 6 countries). Equivalence of care is stated in strategic documents and mostly implemented in 20 countries, while it is stated but not really implemented in 2 countries. It is not stated and not implemented in 1 country. It is stated but partly implemented in 2 countries (no information: 5).

## Discussion

A range of harm reduction interventions and responses to infectious diseases that have been proven to be effective in the community are also available in prisons in Europe. These are implemented in the three stages of incarceration: upon entry, during imprisonment and upon release with referral to services in the communities; however, actual access and coverage remain critical issues and show great differences across countries and by intervention.

Our findings show that while certain essential harm reduction interventions and responses to infectious diseases are officially available in the majority of countries, including screening for drug-related problems upon entry, OST, vaccination, testing, counselling and treatment of infectious diseases, condom distribution, there is great variation in terms of coverage and mode of offering these services throughout the prison stay. Coverage of HIV/HCV/HBV testing is reported to be low in half of the countries and in most cases, it is only offered upon entry to the prison system instead of through all 3 stages: upon entry; during imprisonment and upon release. While OST is available in all but one country, and studies underpin that a large part of people in prison have had problems related to their opioid use [83, 84], this intervention is only available for a minority of people in need, assessed as low coverage in half of the countries—and often only in continuation from the community. Condom distribution coverage—in terms of prisons covered in a country—is also reported to be low in half of the countries. The proportion of people in prison reached by HIV-related health promotion and health education on drug-related infectious diseases are above 30% in the majority of countries, still, some report it to be under this threshold.

Nonetheless, it is shown by our study that various interventions such as PNSPs, disinfectant distribution, lubricant distribution, counselling on safer injecting and risks of tattooing and piercing are only available in a very limited number of countries and often with low coverage or only in few prisons within a country.

There are efforts in the majority of countries to provide linkages to community addiction and HIV, HCV care for those who are in need of such services; however, the level of availability, the mode and the content of referral services vary between countries. Specific upon-release interventions—such as OST initiation before release, take-home naloxone upon release, health education upon release or HIV,HCV, HBV testing upon release are rarely provided that could prepare people—and particularly those who inject drugs—to return to the community and reduce their own health risks and of the people in their social networks.

In the time of our survey, one-third of the countries reported that the ministry responsible for health in a given country is also responsible for prison health, whose structure is probably more effective in integrating prison and community health services and improve the continuity of care provided for people in prison; ministry of health’s responsibility is also fostered by the World Health Organization’s Health in Prisons Programme initiative [55, 85, 86]. In the meantime, in most of the countries, external service providers are involved in providing harm reduction services inside prisons which can facilitate linkages to addiction care in the community upon release. While drug-related interventions in prisons are mentioned in national strategic documents in most of the countries, harm reduction in prison is specifically addressed in 21 countries, while interventions upon release is highlighted in 12 countries only. While ‘equivalence of care’ and ‘continuity of care’ are included in national-level strategic documents in two-third of the countries real-life data measured by our survey and also information provided by the countries suggest that they are often implemented only partially.

The availability and provision of harm reduction interventions in prison remain limited and partly significantly below the level of provision of the same interventions in the community. Some interventions—the effectiveness of which are supported by evidence and are largely implemented in the community—are still scarcely introduced. It must be noted that implementation of such services can still be set back by various prison setting-specific obstacles, such as security, overall ban on illicit drug use inside prisons, lack of capacity, adequate resources, technical expertise, infrastructure and trained staff, attitude towards harm reduction in the prisons such as the peculiar prison context as place of punishment and the moral considerations around it [79, 87–91]. Moralistic attitude to health in prison should be replaced by pragmatic and scientific evidence-based approach to have a public health impact.

Therefore, beside the individual level of helping those in need and improve their health and social well-being and ensuring their right to health [92], harm reduction interventions during imprisonment should be considered as an unmissable public health opportunity [34, 37, 54, 93]. During incarceration, it becomes easier to contact, test and treat otherwise hard to reach risk groups such as PWID with high levels of health-related problems and risks who later return to the community. Consequently, reaching out, diagnosing and treating them in prison also improve the health of their communities after their release, which is called the ‘community dividend’ by Moore [54, 94]. Besides public health gains, addressing drug problems during imprisonment can also help to reduce reoffending among people with drug problems having committed acquisitive crimes which leads to societal benefits as well [95].

In terms of Hepatitis C and B—diagnosis and treatment have become even more feasible and crucial for vulnerable groups such as people who inject drugs and people in prison due to the introduction of highly effective direct-acting antiviral (DAA) therapy, coupled with the 2016 WHO Global health sector strategy on viral hepatitis and the Action plan for the health sector response to viral hepatitis in the WHO European Region [96–98]. These politically approved high-level documents set the target to eliminate viral hepatitis by 2030 for which prisons can act as core settings due to high HCV prevalence among its population and frequent imprisonment of PWID which group carries the highest burden of HCV infection among all risk groups in Europe [99, 100].

Our research, however, is subject to several limitations. As for sources of information the Reitox National Focal Points’ workbooks are the best available, as they contain thematic, nationally consolidated and scrutinised information in English in a format, which is harmonised across countries; still the reported data on interventions have limited comparability across countries, especially regarding coverage and mode of implementation due to lack of information and unified data collection methods at national level.

Countries’ data source selection per specific sub-topics and publication policies may differ: they are collating information from different sources at national level, such as public administration reports, prison registry data, scientific literature of well-designed studies or independent research and grey literature or expert opinions, the quality of which cannot yet be controlled at European level. In some countries, newer scientific sources may be available, however not yet reported by the National Focal Point for authorisation issues. Regional or local differences or variability due to correction facility types or inmate groups can make the picture of harm reduction availability puzzling even within one country. Thus, heterogeneous national data from the different countries were collided to a common set of variables in order to make 30 standardised country profiles. Some collateral data losses and simplification could certainly occur during the process.

Regarding the questionnaire survey that aimed to fill the gaps of the desk research phase also conducted among National Focal Points carries the same limitations in terms of data source variations across countries that were used for answering the questions. Nevertheless, National Focal Points based their answers on the best nationally available sources and committed to provide the least biased, most objective estimate possible. If no survey or routine data were available, expert estimates provided by NFP staff on coverage for example were usually triangulated among different national data sources and information.

As aggregate data was collected per country for all types of prisons, distinguishing interventions per type of prisons could not be made in this current analysis. However, as pre-trial detainees form a considerable proportion of all people in prison and their situation and needs may be different, it is recommended that future studies should aim to separate harm reduction services available to this group from those available to sentenced people.

Important to mention that this paper is based on the data collected through the HAREACT project and referred to a specific period (2016/2017) since then data may have changed in the meantime because of actual change in the situation.

It is noteworthy that quality assurance, effectiveness or outcome evaluation of prison-based interventions in prison could not be covered in the mapping process due to the lack of information, which is indeed a normal practice in community prevention, treatment and harm reduction interventions. However, it is questionable whether specific evidence is needed for prisons, if the same community interventions are backboned by robust evidence; also, the community dividend public health approach benefits of prison-based interventions are apparent [51, 54, 94].

Our focus was on PWID in prison and on interventions responding to risks and problems related to injecting and infectious diseases. However, it must be noted that while people who inject opioids can be addressed by various interventions, stimulant and new psychoactive substance use including injecting are also present phenomena in the prison population [15, 101, 102], but a limited number of harm reduction interventions can respond to their needs [103].

Our findings call for attention of further monitoring efforts and sustainability. The definition of availability of harm reduction interventions has many aspects that may mask significant differences between the countries and extent of service provision (formal availability, actual availability, coverage, and quality of interventions). Public administration may call a service available on the basis of legal context, which does not necessary mean real availability. Information available on drug-related interventions—especially regarding coverage, content and regularity—is still scarce in general in the EU-30 and the available information is limitedly comparable across countries. Information on coverage is limited in the two dimensions: prisons covered and people covered. However, recent efforts are ongoing at European level to improve the data availability and cross-country comparability through the implementation of a European monitoring framework on drugs and prison and piloting of a European model facility survey questionnaire on drug-related interventions in prisons that would be available in the coming period [104]. Despite several gaps in monitoring and quality, our analysis provides a comprehensive and updated overview on harm reduction interventions in European prisons; the presented data inform international, national and local policy makers and service planners to improve responses for people in prison with drug-related problems, providing direct public health benefits.

## Conclusions

As the benefits of drug-related harm reduction interventions for individuals and public health are widely documented, these interventions should be accessible to people who use drugs in community as well as in prison settings. Despite limitations in obtainability and comparability of information from European prison systems, our study allows us to conclude that availability of, and access to harm reduction services in European prisons is highly variable between countries and in general lower than in the community. Even for harm reduction measures that are well established in the community, we noted significant delays in their introduction in prisons. When interventions are offered in prison, their coverage is low and the quality of implementation lags behind. There is a gap between international recommendations and ‘on-paper’ availability and the actual implementation of interventions. Scaling up harm reduction measures in prison can achieve important individual benefits and results in additional dividends for public health. Our study also points to the need to improve prison system’s documentation of responses to health harms and to increase the comparability of information and data to inform programme planning and policy making at national and international level.

## Data Availability

The comprehensive report entitled ‘European Mapping of Harm Reduction Interventions in Prisons’ (EMR) including the datasets generated within the HA-REACT Joint Action project was analysed during the current study that are publicly available in the harmreduction.eu repository: https://harmreduction.eu/documents/Mapping_Report_rev_July2019.pdf.

## Abbreviations

DRID: Drug-related infectious diseases
ECDC: European Centre for Disease Control and Prevention
EMCDDA: European Monitoring Centre for Drugs and Drug Addiction
EMR: European Mapping of Harm Reduction Interventions in Prisons
EU-30: 27 Member States of the European Union, Norway, Turkey and the United Kingdom
HA-REACT: Joint Action on HIV and Co-infection Prevention and Harm Reduction
HBV: Hepatitis B virus
HCV: Hepatitis C virus
HIV: Human immunodeficiency virus
IP: Imprisonment
NFP: National focal point
NSP: Needle and syringe programme
OST: Opioid substitution treatment
PEP: Post-exposure prophylaxis
PNSP: Prison-based needle and syringe programme
PWID: People who inject drugs
SB: Statistical Bulletin of the EMCDDA
SPACE: Council of Europe Annual Penal Statistics project
SQ: Structured Questionnaire for reporting to the EMCDDA
ST: Standard Table for reporting to the EMCDDA
TB: Tuberculosis
UNODC: United Nations Office of Drugs and Crime

## Footnotes

1 Reitox is the European information network on drugs and drug addiction created at the same time as the European Monitoring Centre for Drugs and Drug Addiction. The abbreviation ‘Reitox’ stands for the French ‘Réseau Européen d ´Information sur les Drogues et les Toxicomanies’. The Reitox network as integral part of the EMCDDA included at the time of the survey the 27 Member States of the European Union, Norway, Turkey, the UK and the European Commission. The network is a cornerstone of the European drug monitoring and reporting system, to which member states contribute among others by collecting, analysing, interpreting national drug-related data systematically based on standardised common methodological guidelines, reporting and analytical tools. See at: https://www.emcdda.europa.eu/publications/posters/2017/reitox-development-framework_en, https://www.emcdda.europa.eu/system/files/publications/12032/Reitox%20FAQs%20Oct2019_FINAL.pdf.

2 The Joint Action on HIV and Co-infection Prevention and Harm Reduction (HA-REACT) was a three-year project funded by the Health Programme 2014–2020 from European Union between 2015 and 2018. It aimed to identify gaps in the realisation and scale-up of harm reduction measures to prevent HIV and other co-infections, especially tuberculosis and viral hepatitis, among people who inject drugs. The scope of work package 6 was harm reduction and continuity of care in prisons (ID: GA# 677085).

3 In the meantime, the UK has left the EU and subsequently EMCDDA membership.

4 This information is out of the reporting period of our mapping research; however, authors considered it important to update this information with 2018 data retrieved from EMCDDA. All other OST-related variables refer to 2016/2017.

5 No information from Scotland and Wales.

6 It is called proxy as the denominator is all people in prison during reporting year instead of people in need in prisons during the reporting year, as the latter is not available thus it is not sensitive data in terms of the level of demand.

7 UK: only England and Wales provided information.

8 Hungary data was corrected compared to the HAREACT report. UK: Wales and England—full coverage, Northern Island no coverage, Scotland no data on coverage—was listed as full coverage country.

9 UK: reported only by Wales and England.

10 UK was counted as medium coverage country; however, England reported low coverage, while Wales and Scotland medium coverage (no information was available about Northern Ireland).

11 Northern Ireland reported 10%; however, in this description only Wales and England are presented.

12 The UK was counted as low coverage country. England, Scotland and Northern Ireland reported low coverage, while Wales reported medium coverage.

13 UK only represents in this category Northern Ireland.
